# The glioblastoma ecosystem: clonal evolution, heterogeneity, and therapeutic resistance

**DOI:** 10.3389/fcell.2026.1834106

**Published:** 2026-07-01

**Authors:** Ying He, Wenxue Song, Shuo Li, Jing Guo

**Affiliations:** 1 The Sixth Affiliated Hospital of Harbin Medical University, Harbin, Heilongjiang, China; 2 Harbin Medical University Affiliated Cancer Hospital, Harbin, Heilongjiang, China

**Keywords:** clonal evolution, glioblastoma, glioma stem cells, single-cell sequencing, spatial transcriptomics, therapeutic resistance, tumor heterogeneity

## Abstract

Therapeutic resistance and recurrence represent major clinical challenges in glioblastoma (GBM), driven by profound tumor heterogeneity and continuous clonal evolution under therapeutic pressure. Conventional diagnostic and therapeutic strategies, which rely on static sampling, struggle to effectively address this dynamic ecosystem. This review synthesizes recent evidence on how single-cell and spatial multi-omics technologies are uncovering the multidimensional complexity of GBM, spanning its diverse cell states, spatial architecture, and clonal dynamics. We dissect the core mechanistic networks driving this evolution, including genomic instability, microenvironmental selection pressures, cellular plasticity, and the integrative role of core signaling pathways. Furthermore, we critically examine the limitations of static diagnostics and propose the pathways through which heterogeneity mediates therapeutic resistance. Given these challenges, future clinical management should ideally transition from a static classification to a dynamic precision paradigm. To this end, we explore the application prospects of dynamic monitoring technologies based on liquid biopsy and radiomics, as well as novel therapeutic strategies aimed at targeting the evolutionary process itself. Ultimately, reconceptualizing GBM as a dynamically evolving ecosystem provides a foundational framework for understanding therapeutic resistance and is pivotal for developing novel strategies that target the evolutionary process itself. However, the clinical translation of this framework faces significant hurdles, including the restrictive blood-brain barrier, technical constraints in longitudinal monitoring, and the complex signaling redundancies that necessitate more adaptive, evolution-informed clinical trial designs. This review suggests a potential path toward a new paradigm of dynamic precision medicine.

## 1 Introduction

Glioblastoma (GBM) remains the most aggressive primary malignant tumor of the central nervous system, characterized by a trajectory of recurrence and therapeutic resistance (Chang et al., 2024). Despite standard-of-care protocols involving maximal safe surgical resection followed by temozolomide-based concurrent chemoradiotherapy, the vast majority of tumors recur, often at the peritumoral brain zone (Moujalled et al., 2022). This dismal prognosis is largely attributable to the high likelihood of recurrence and the tumor’s manifestation of both intrinsic and acquired resistance to multiple therapies. Most patients experience disease progression following initial treatment, with recurrent tumors typically exhibiting increased aggressiveness and therapeutic insensitivity, ultimately leading to therapeutic failure. This reality underscores the necessity of a shift beyond traditional, static views of the tumor to explore the deeper biological complexities underlying these behaviors. For a long time, the diagnosis and classification of GBM have relied on the World Health Organization (WHO) grading system and molecular subtypes—such as classical, mesenchymal, and proneural—provided by The Cancer Genome Atlas (TCGA) (Alnahhas, 2024). While these classifications have significantly enriched our understanding of inter-tumoral heterogeneity, their static nature may fail to capture the dynamic evolution and subtype transitions that GBM undergoes under therapeutic pressure, revealing a fundamental limitation of this perspective.

**FIGURE 1:**
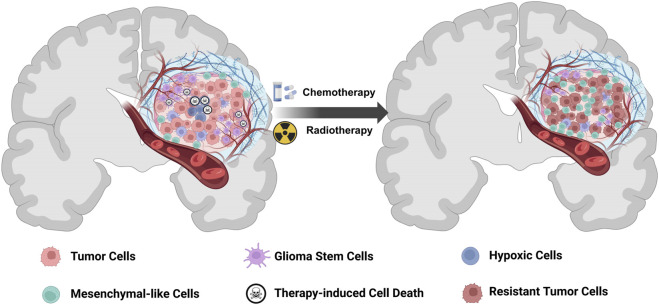
The dynamic evolution of the glioblastoma ecosystem under therapeutic pressure. Original schematic illustrating the Darwinian selection process within the GBM ecosystem during standard chemoradiotherapy. Before treatment (left), the tumor exhibits high intratumoral heterogeneity, containing tumor cells, glioma stem cells (GSCs), and hypoxic cell niches. Following therapeutic intervention (chemotherapy and radiotherapy), the selective pressure eradicates treatment-sensitive populations, facilitating the expansion of resistant subclones, particularly mesenchymal-like cells and GSCs (right), which ultimately drive disease recurrence. Abbreviations: GSCs, glioma stem cells; GBM, glioblastoma.

Consequently, the research paradigm is fundamentally shifting from static description to dynamic evolution. This paradigm centers on conceptualizing GBM as an “ecosystem,” which is characterized by intratumoral heterogeneity and continuous clonal evolution (Kampers et al., 2025). Intratumoral heterogeneity exists not only in the spatial dimension—where distinct regions of the same tumor harbor cell subpopulations with diverse genotypes and phenotypes (spatial heterogeneity)—but more critically, in the temporal dimension. Tumor cells undergo continuous Darwinian evolution over the disease course, particularly under the selective pressure of treatment (temporal heterogeneity) (Mathur et al., 2024; Couturier et al., 2020). This process, known as clonal evolution, results in the selective enrichment of cellular subclones with survival advantages, which eventually become the dominant population in recurrent tumor (Favero et al., 2015). Within this context, the scope of this review is to map the multidimensional architecture of the GBM ecosystem, specifically focusing on the interplay between intrinsic cellular plasticity and extrinsic therapeutic pressures. We systematically delineate the driving mechanisms of heterogeneity and clonal evolution in GBM, argue for the necessity of transitioning from static classification to dynamic precision management, and provide a perspective on novel diagnostic and therapeutic strategies based on evolutionary biology principles (Figure 1).

## 2 Methods

This review followed a structured approach to synthesize recent advances in GBM tumor heterogeneity and clonal evolution. We performed a comprehensive literature search across the PubMed/MEDLINE and Web of Science databases, with the last search conducted on 1 May 2026. To ensure a balanced and high-quality evidence base, our search strategy incorporated a combination of Medical Subject Headings (MeSH) and free-text keywords (“glioblastoma” OR “GBM”) AND (“tumor heterogeneity” OR “clonal evolution”) AND (“single-cell sequencing” OR “spatial transcriptomics”) AND (“tumor microenvironment” OR “therapeutic resistance”). The search was restricted to articles published between 2015 and 2026. The systematic screening and selection process is illustrated in the PRISMA flow diagram (Supplementary Figure S1). In terms of study selection, we prioritized peer-reviewed original research, authoritative reviews, and landmark studies that collectively illustrate the multidimensional nature of the GBM ecosystem. Inclusion criteria focused on studies providing biological or clinical insights into GBM heterogeneity. Studies were excluded if they were case reports, preprints, non-English publications, or not specifically focused on GBM. Key studies identified from reference lists of retrieved articles were also integrated to ensure comprehensive coverage.

## 3 Results

### 3.1 Unraveling multidimensional heterogeneity: from technological breakthroughs to bio-logical insights

Understanding the mechanisms of therapeutic resistance in GBM requires deciphering its intrinsic heterogeneity. Recent advances in single-cell and spatial multi-omics technologies have enabled researchers to move beyond traditional bulk tissue-based analyses, systematically deconvolving the complex composition of GBM across cellular and spatial dimensions. In the following sections, we elaborate on the features and biological implications of GBM heterogeneity in spatial organization and temporal dynamics, as revealed by these technological advancements.

#### 3.1.1 Single-cell and spatial multi-omics technologies: core tools for deconvoluting complexity

Conventional bulk tissue-based genomic and transcriptomic analyses average the molecular signals of all cells within a tumor, often obscuring the underlying cellular diversity. To overcome this, single-cell and spatial multi-omics technologies enable a high-resolution dissection of the GBM ecosystem by performing molecular analyses on individual cells while preserving their native spatial context.

Single-cell RNA sequencing (scRNA-seq) provides an unbiased view of the cellular panorama within the tumor microenvironment (TME), revealing continuous spectra of transcriptional states—including neural-progenitor-like, mesenchymal-like, and oligodendrocyte-progenitor-like states (Hara et al., 2021). This suggests that malignant cell identity in GBM is highly plastic, frequently undergoing dynamic transitions in response to microenvironmental cues rather than being rigidly fixed. Complementing this, single-cell DNA sequencing (scDNA-seq) identifies single nucleotide variants and copy number alterations, enabling the reconstruction of phylogenetic trees to elucidate clonal origins and evolutionary relationships (Zhang et al., 2023; Weber et al., 2023). When integrated with spatial technologies, such as spatial transcriptomics and multiplexed immunofluorescence, these tools anchor molecular insights to specific histological niches, such as perivascular regions, necrotic peripheries, and the invasive front (Yang et al., 2024; Onubogu et al., 2024).

The genomic complexity of GBM is thus not confined to static mutational landscapes but manifests as an intricately intertwined network of inter-clonal genetic heterogeneity and transcriptional plasticity, driven by the interplay between stochastic mutations, therapy-induced genomic instability, and adaptive state transitions. By leveraging these multi-layered technologies, research has demonstrated that GBM often exhibits a branched evolutionary pattern, with multiple genetically distinct subclones already present at diagnosis (Sottoriva et al., 2013). Furthermore, these technologies reveal a strong correlation between cellular states and spatial context; for instance, mesenchymal-like cells are frequently enriched at the invasive front, whereas stem-like cells predominantly reside in perivascular niches (Ghochani et al., 2022).

**FIGURE 2:**
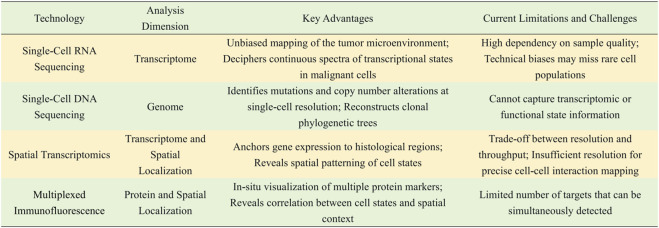
Comparison of single-cell and spatial multi-omics technologies for deconvoluting GBM complexity. Summary of the key analytical dimensions, technological advantages, and current methodological limitations for major single-cell and spatial techniques utilized in GBM research. This comparison highlights the complementary nature of these approaches in mapping the tumor ecosystem, from genomic phylogenetics to spatial histological localization. Abbreviations: GBM, glioblastoma.

Despite these advancements, applying these technologies faces challenges including sensitivity to sample quality, trade-offs between resolution and throughput, and the significant computational demands of multi-dimensional dataset integration. While these represent active areas of methodological research, they do not diminish the novel insights into GBM heterogeneity that these technologies have provided. A summary of these capabilities and limitations is provided in Figure 2.

#### 3.1.2 A dynamic ecosystem forged by spatial and temporal heterogeneity

The spatial organization of GBM is not a static backdrop but a dynamic stage that facilitates clonal evolution. This section connects the previously described cellular states to their geographical niches and reveals how their interplay in space and time forges the evolving ecosystem that drives recurrence. Multi-omics findings reveal that GBM is not a disorganized cellular aggregate but a system with a highly organized architecture (Read et al., 2024; Liu M. et al., 2024). The diverse core cellular states defined by single-cell transcriptomics exhibit distinct regional enrichments within the tumor, forming the basis of spatial heterogeneity. Hypoxic regions in the tumor core are predominantly populated by cell clusters expressing stress-response and autophagy-related genes, whose survival hinges on adaptive responses to hypoxic conditions (Lemoine et al., 2025).

This spatial organization is directed by highly specialized molecular programs. For instance, in the hypoxic core, the stabilization of HIF-1α activates transcriptional networks that upregulate genes involved in metabolic reprogramming, such as P4HA1 (18), thereby ensuring cellular survival under low-oxygen stress. Furthermore, temporal clonal dynamics are underpinned by distinct genetic drivers. The amplification of EGFR (particularly the EGFRvIII variant) acts as a primary engine for proliferative signaling, yet this driver can be bypassed under therapeutic pressure by the compensatory activation of the MET or PI3K/AKT pathways (Singh et al., 2021). Simultaneously, the loss of tumor suppressors such as PTEN creates a permissive environment for genomic instability, facilitating the emergence of diverse subclonal lineages that are genetically equipped to withstand standard therapies (Brennan et al., 2013; Minea et al., 2024). Collectively, these specific genetic alterations and their downstream signaling interactions orchestrate the adaptive trajectories of the GBM ecosystem.

In contrast, the invasive front is chiefly composed of cells with a mesenchymal-like transcriptional state, characterized by high expression of genes like ZEB1 and STAT3 involved in extracellular matrix remodeling and cell migration (Hendriksen et al., 2024; Lv et al., 2022); their spatial distribution strongly correlates with the infiltrative growth pattern (Verhaak et al., 2010; Bhat et al., 2013). Furthermore, the perivascular niche is recognized as a key reservoir for glioma stem cells (GSCs), where interactions with stromal cells, such as endothelial cells and tumor-associated macrophages, collaboratively maintain a microenvironment conducive to stemness maintenance and therapeutic resistance (Calabrese et al., 2007).

This highly organized spatial structure provides the stage for clonal evolution. Temporal heterogeneity—the clonal dynamics under therapeutic pressure—is inextricably intertwined with the spatial dimension, collectively shaping a dynamically evolving ecosystem (Goenka et al., 2021; Qazi et al., 2022). Therapeutic resistance and recurrence in GBM are direct consequences of clonal succession and adaptive evolution of the tumor cell population within this spatial framework. The evolutionary patterns primarily manifest in three forms, each closely linked to spatial structure: First, evolution occurs as “regional parallel evolution.” Subclones residing in geographically separated niches (e.g., tumor core versus invasive front) within the primary tumor independently acquire genetic or epigenetic alterations under therapeutic selection (Onubogu et al., 2024; Kim et al., 2015). The prevalence of branched evolution is a structural hallmark of GBM. Quantitative analysis in seminal multi-region sequencing cohorts revealed that branched evolutionary patterns occur in approximately 85% of cases, with multiple genetically distinct subclonal lineages detectable as early as initial diagnosis (Qazi et al., 2022). This model is further supported by a recent three-dimensional whole-tumor sequencing study, which visualized the macroscopically segregated architecture of subclones across different tumor masses, providing direct spatial evidence for regional parallel evolution (Mathur et al., 2024). Concurrently, evolution often manifests as “clonal decline and resurgence.” Therapy eradicates treatment-sensitive dominant clones, thereby freeing up ecological space for pre-existing yet quiescent or minor “founder clones” to expand. This allows such clones to “re-emerge” from their original or ectopic locations and become the major population at recurrence (Goenka et al., 2021; Kim et al., 2015). Furthermore, “clonal migration and colonization” is a critical pattern. Subclones with specific survival advantages migrate from their native niche to other regions and successfully establish new tumor sites, acting as seeds for distant recurrence (Johnson et al., 2014).

These three patterns collectively demonstrate a complex interplay between spatial heterogeneity and evolutionary dynamics. The standard therapies themselves are likely the key selective pressures driving this evolutionary process. For instance, longitudinal single-cell studies have quantified the impact of standard-of-care treatment on tumor cell identity. While inter-tumoral heterogeneity leads to variable clinical outcomes, representative cohorts have demonstrated that the proportion of cells exhibiting a mesenchymal-like transcriptional state can increase from approximately 25% at primary diagnosis to over 60% in recurrent tumors following chemotherapy and radiotherapy (Matsumoto et al., 2020). This shift is not merely a consequence of clonal selection; temozolomide and radiotherapy also induce DNA damage and promote hypermutator phenotypes (Hegi et al., 2005; Vitiello et al., 2025). Similarly, radiotherapy, while eliminating sensitive cells, can alter the microenvironment to create favorable conditions for the “resurgence” or “migration and colonization” of specific clones (Zhang et al., 2018; Hart et al., 2024). These evolutionary patterns reflect the dynamic adaptation of the tumor cell population under the potent selective pressure exerted by treatment.

Moving beyond descriptive accounts of evolutionary history towards predicting its future trajectory, the field is increasingly incorporating mathematical models from evolutionary biology. For example, branching process models can describe how tumor-initiating cells form a population comprising multiple genetic subclones through stochastic processes of proliferation, death, and acquisition of driver mutations (Caravagna et al., 2020). When accounting for the marked spatial structure of GBM, spatial evolutionary models further incorporate the effects of cell migration and local resource competition to simulate clonal dynamics across functional regions. Additionally, evolutionary game theory provides a powerful theoretical framework for understanding the interactions between clones mediated by secreted factors or resource competition (Winkler, 2023). Integrating these models with longitudinal molecular data holds the promise of shifting from “*post hoc* explanations” to “proactive predictions” of tumor evolutionary trajectories. In summary, the profound heterogeneity of GBM is a systematic process driven by a dual engine: long-term evolutionary potential rooted in pre-existing genetic diversity and rapid adaptive capacity fueled by cellular plasticity. The synergy between these mechanisms shapes the spatiotemporal complexity we observe, and deciphering this system is essential for developing effective intervention strategies.

### 3.2 Core mechanisms driving heterogeneity and clonal evolution

The profound heterogeneity and evolution described above are orchestrated by a multi-layered functional architecture. This chapter aims to propose an integrative framework positing that the core mechanisms driving this process do not operate in isolation but constitute a hierarchical functional architecture. This system is underpinned by genomic instability providing the substrate for diversity, shaped by external selection from the TME, mediated by rapid adaptation via cellular plasticity, and ultimately integrated and executed by core signaling networks.

#### 3.2.1 Genetic foundation: genomic instability drives clonal diversity

Genomic instability provides the genetic substrate for intratumoral heterogeneity in GBM. Chromosomal instability is a primary manifestation of this phenomenon. It leads to widespread aneuploidy and copy number alterations, which in turn drive key events such as EGFR amplification and PTEN deletion (Brennan et al., 2013; Liu Y. et al., 2024). Crucially, therapeutic intervention itself exerts a potent genome-perturbing effect. The enrichment of temozolomide-induced mutational signatures in recurrent tumors provides direct evidence that treatment actively shapes tumor evolution (Minea et al., 2024). This process is notably exacerbated by mismatch repair deficiency, which can foster a hypermutator phenotype, supplying a nearly limitless reservoir of genetic diversity (Hadad et al., 2023; Pu et al., 2025).

Mechanistically, the interplay between therapeutic-induced DNA damage and defective cell cycle checkpoints is a critical driver of this hypermutator phenotype. Research has elucidated that the failure of DNA damage repair (DDR) mechanisms——often involving dysregulation of key kinases such as PLK1, CHK1, and CHK2—allows cells to bypass vital checkpoints and re-enter the cell cycle with unrepaired lesions (Verma et al., 2019). This process not only facilitates immediate survival but also imposes a substantial mutational tax on the genome, directly generating novel genomic diversity that fuels clonal evolution.

Beyond direct DNA damage, genomic instability relies on dysregulated downstream molecular circuits. For instance, the frequent inactivation of ARID1A, a component of chromatin remodeling complexes in GBM, alters genome-wide chromatin accessibility. This impacts DNA repair efficiency and permits the aberrant activation of silent oncogenes, providing an epigenetic basis for cell state transitions (Bakr et al., 2024). Furthermore, dysregulated telomere maintenance—via either TERT reactivation or the alternative lengthening of telomeres (ALT) pathway—is pivotal for GBM immortality and its sustained tolerance of genomic instability. Collectively, these mechanisms ensure a continuous supply of raw material for clonal evolution under persistent therapeutic pressure. Consequently, a mutually reinforcing relationship exists: cytotoxic therapy not only selects for pre-existing resistant clones but also actively contributes to tumor evolution by inducing new genomic alterations, necessitating a balance between immediate therapeutic efficacy and the long-term risk of fostering further diversification.

#### 3.2.2 External selection: the TME shapes clonal evolutionary trajectories

The TME is a dynamic ecosystem that continuously selects for fitter cell populations through the concerted action of biophysical and chemical factors. Rather than acting as a static soil that passively selects for seed cells, the TME is a co-evolving participant that constantly updates the fitness landscape of the GBM ecosystem. For instance, the hypoxic niche, by stabilizing hypoxia-inducible factors (HIFs), activates transcriptional programs involved in stemness maintenance and metabolic reprogramming. Recent research highlights that this metabolic adaptation, particularly the altered branched-chain amino acid metabolism regulated by HIFs, provides a distinct survival advantage to tumor subpopulations (Zhu et al., 2021; Zhang et al., 2021).

Concurrently, the TME orchestrates a sophisticated immunological crosstalk that steers evolutionary trajectories. Immune editing serves as a crucial selective force, progressively enriching for clones equipped with immune-evasion capabilities, such as defects in antigen presentation and the upregulation of immune checkpoints (Long et al., 2025; Espinoza et al., 2025). Current advances indicate that diverse components of the TME collectively shape the tumor evolutionary trajectory by establishing a network of synergistic interactions. For instance, hypoxic regions not only directly select for hypoxia-tolerant clones but also secrete cytokines that remodel immune cell functions, fostering a localized immunosuppressive niche that indirectly facilitates the expansion of immune-evading clones (Lu et al., 2024; Lin et al., 2024). Furthermore, signaling molecules such as TGF-β, secreted by tumor-associated macrophages among others, act as key inducers driving the mesenchymal transition of tumor cells while simultaneously suppressing anti-tumor immunity (Cui et al., 2018). Therefore, the TME actively shapes the adaptive phenotypes of tumor cells, rather than merely passively selecting dominant clones.

#### 3.2.3 Rapid adaptation: cellular state plasticity mediates non-genetic therapy resistance

Cellular state plasticity, the ability of cells to undergo dynamic transitions between functional states, is central to mediating non-genetic therapy resistance in GBM. The cellular basis for this plasticity lies in the multi-lineage differentiation potential of glioma stem cells (GSCs), which act as a functional reservoir capable of generating transcriptionally distinct progeny to facilitate phenotypic adaptation. Longitudinal single-cell transcriptomic studies have revealed that standard treatments, including radiotherapy and temozolomide, induce a systematic temporal reprogramming of the tumor landscape, typically characterized by a marked enrichment of the mesenchymal-like state (Wang et al., 2022). This transition is highly adaptive, as the mesenchymal phenotype is strongly associated with enhanced invasive capacity, angiogenesis, and resistance to cytotoxic stress (Hendriksen et al., 2024; Matsumoto et al., 2020; Chandra et al., 2020).

The realization of this plasticity depends on a highly dynamic transcriptional and epigenetic regulatory network. Core transcription factors (e.g., OLIG2, SOX2) define distinct cellular states by forming specific protein interaction networks and cooperating with chromatin-modifying enzymes (e.g., EZH2, HDACs). For instance, the transition from a proneural to a mesenchymal state involves not only the activation of signaling pathways like NF-κB/STAT3 but is also coupled to a genome-wide remodeling of histone modifications (Hara et al., 2021; Loftus et al., 2024). Furthermore, this adaptive capacity is intrinsically linked to checkpoint adaptation; when cells are forced to resolve DNA damage through compromised signaling, they may undergo cell cycle re-entry despite persistent double-strand breaks. The constitutive activation or dysregulation of molecules such as PLK1, CHK1, and CHK2 marks this state, providing a transient window for non-genetic phenotypic switching that often precedes the stabilization of permanent genetic alterations (Verma et al., 2019). These coordinated changes collectively drive a systematic reprogramming of the cellular transcriptome. Non-coding RNAs act as critical regulatory nodes in this process, forming intricate circuits with transcription factors and chromatin complexes to finely tune the kinetics of state transitions. Consequently, targeting these regulatory nodes is envisioned as a potential strategy to “lock” cells in a specific state and reverse resistant phenotypes.

#### 3.2.4 Systems integration: core signaling pathways coordinate multidimensional driving forces

The aforementioned genetic diversity, microenvironmental pressures, and cellular plasticity constitute the three fundamental driving forces of GBM evolution. Crucially, these forces do not operate in isolation but are integrated and executed through a highly interconnected core signaling network. This network not only receives input signals from the genome, microenvironment, and intracellular components but also actively drives clonal evolution by regulating downstream effectors. This integrative role is first evident in how the network interprets and executes genetic instructions. Specific genetic alterations within the RTK/RAS/PI3K signaling axis show significant correlations with distinct cellular states (Lv et al., 2022; Verhaak et al., 2010), establishing a direct molecular link within the genotype-phenotype continuum. Conversely, the inactivation of the p53 and RB pathways creates a permissive internal environment for the accumulation of genomic instability and the exploration of cell states by disrupting critical cell cycle checkpoints and apoptotic responses (Crespo et al., 2015; Eisenbarth and Wang, 2023). Simultaneously, this network efficiently executes the selective pressures originating from the microenvironment. For instance, the TGF-β pathway acts as a bridge connecting the microenvironment to cellular phenotype, not only mediating immunosuppression but also potently driving the mesenchymal transition of tumor cells and the activation of associated therapy-resistant programs (Setlai et al., 2022; Lai et al., 2024). More importantly, this signaling network directly enables cellular state plasticity. Under therapeutic pressure, pathways such as TGF-β and RTK cooperate with inflammation-related pathways like NF-κB and STAT3 to instruct cells to switch between different functional states (e.g., towards a mesenchymal-like state) by regulating core transcription factors and epigenetic remodeling, thereby mediating rapid phenotypic adaptation (Shi et al., 2023; Garner et al., 2013).

These pathways achieve their systems-level integrative function through complex crosstalk and feedback loops, which remodel downstream protein-protein interaction networks and gene regulatory programs. A key example is the EGFRvIII mutant, which demonstrates preferential activation of the PI3K-AKT and STAT pathways over the ERK pathway through its ability to assemble into distinct spatial complexes with specific adaptor proteins and kinases (Mahajan and Mahajan, 2015; Cooper et al., 2022). This biased signaling output originates from mutation-induced alterations in protein interaction interfaces. Furthermore, pathway activity directly orchestrates the expression of thousands of genes via transcription factors and epigenetic regulators, establishing a highly interconnected gene regulatory network. Critical network analyses reveal that rearranged super-enhancers in GBM are associated with the coordinated, high-amplitude transcription of core cell identity genes. This positions super-enhancers as a pivotal mechanism for integrating upstream genetic alterations and signaling pathway activities, thereby facilitating the stable maintenance of specific cellular states (Xie et al., 2024). Deciphering this comprehensive signaling cascade—from membrane receptors to chromatin remodeling—is therefore essential for elucidating genotype-phenotype relationships and developing effective combination therapeutic strategies.

In summary, the heterogeneity and evolution of GBM constitute a systems-level problem driven by multi-layered mechanisms—genetic, phenotypic, and microenvironmental—integrated via a core signaling network. These pathways do not operate linearly but form a dynamic and robust regulatory network through intricate crosstalk and feedback loops. Understanding the topology and dynamics of this network is crucial for predicting tumor evolutionary trajectories and designing combination therapies capable of effectively disrupting its systemic stability. This highly interconnected, systems-level property endows GBM with a powerful buffering and adaptive capacity, inevitably leading to the failure of any static intervention targeting a single molecular node due to compensatory activation within the network. This fundamental characteristic poses significant challenges to the clinical diagnosis and treatment of GBM, while simultaneously pointing the way towards emerging interventional paradigms that target the evolutionary process itself, which will be elaborated in the following sections.

### 3.3 Clinical implications and translational perspectives

The intratumoral heterogeneity and ongoing clonal evolution in GBM, driven by the mechanisms previously outlined, are fundamental biological properties of the disease. These features directly underpin the limited efficacy of standard-of-care protocols and the near-inevitability of disease progression. This chapter systematically delineates the fundamental limitations exposed in both diagnosis and treatment when traditional strategies, built upon a static understanding, confront a dynamically evolving ecosystem. It further outlines the emerging monitoring and therapeutic paradigms developed to address these challenges.

#### 3.3.1 Challenges and innovations in traditional diagnostic models

The conventional pathological diagnostic model relies on single-site biopsy, which suffers from inherent limitations in representativeness when dealing with GBM, a disease marked by profound spatial heterogeneity. Multi-region sequencing has confirmed significant genomic and transcriptomic disparities between different areas of the same tumor (Mathur et al., 2024; Nomura et al., 2025). Such spatial variation can lead to a failure of single-site biopsies to capture key driver mutations or accurately reflect the tumor’s malignant potential, thereby compromising the precision of treatment decisions. For instance, reliance on a single biopsy sample might miss EGFR amplifications present only in specific regions or fail to accurately quantify the proportion of aggressive cellular subpopulations (e.g., mesenchymal-like cells), which are key determinants of treatment response.

To address this challenge, clinical diagnostic approaches are shifting toward multi-dimensional integration. Multi-region sequencing, by systematically analyzing samples from distinct anatomical regions within the tumor, enables the construction of a more comprehensive genomic landscape and reveals the spatial architecture of clonal distribution. Concurrently, image-guided targeted biopsy techniques, leveraging advanced MRI sequences such as perfusion-weighted imaging and diffusion tensor imaging, can identify areas with the highest proliferative activity or invasiveness within the tumor, thereby enhancing the representativeness of the sampled tissue. The future diagnostic paradigm will no longer rely on a solitary pathology report but will instead integrate histopathology, multi-regional molecular profiling, and radiomic features extracted from medical imaging to generate a personalized “tumor heterogeneity map.” This comprehensive profile will provide a precise spatial and molecular foundation for subsequent therapeutic strategies.

#### 3.3.2 Diverse mechanisms of therapy resistance mediated by heterogeneity

Tumor heterogeneity contributes to the biological basis of therapeutic resistance through multiple, parallel, and dynamic pathways. These mechanisms can be categorized into three interconnected themes: primary resistance due to heterogeneous target distribution, acquired resistance driven by therapy-induced evolution, and functional sanctuaries provided by the tumor’s anatomical structure. While different therapies have distinct mechanisms of action, the core principle remains: any therapeutic intervention exerts selective pressure that systematically enriches pre-existing or newly emerged resistant clones, while simultaneously reshaping the tumor ecosystem towards a therapy-resistant state.

First, heterogeneity in target distribution leads to primary resistance. The successful application of both temozolomide chemotherapy and molecularly targeted therapies critically depends on one factor: the ubiquitous expression of their specific molecular targets across the entire tumor cell population. In chemotherapy, the effectiveness of temozolomide is not only limited by the overall MGMT promoter methylation status of the tumor but more crucially, any pre-existing subclones in which MGMT expression is not silenced are selectively enriched during temozolomide treatment, becoming the seeds for resistant recurrence (Singh et al., 2021). Similarly, for targeted agents like EGFR inhibitors, the pre-existence of target-negative subclones or cell populations with activated alternative signaling pathways renders single-agent targeted therapy ineffective against all malignant cells from the outset (Chabon et al., 2016; Gerlinger et al., 2012).

Second, the treatment process itself actively accelerates tumor evolution, fostering acquired resistance. This occurs not only through the selection of existing clones but also by inducing genomic and phenotypic instability. Prolonged temozolomide exposure can induce mismatch repair deficiency, thereby promoting a hypermutator phenotype that vastly expands the genetic diversity available for resistance mutations (Vitiello et al., 2025). Furthermore, radiotherapy and certain targeted agents, acting as potent environmental stressors, are strongly associated with phenotypic switching of tumor cells towards a mesenchymal state—a state itself considered a key driver of therapy resistance due to its association with enhanced DNA damage repair capacity, stemness, and invasiveness (Xiong et al., 2020). Thus, while eliminating sensitive cells, therapy may simultaneously promote the emergence of more adaptable, resistant populations.

Finally, the functional anatomical structure of the tumor provides physical and functional sanctuaries for resistant clones. GSCs are often enriched within specific niches, such as the perivascular region (Li et al., 2009). Their inherent quiescence, robust DNA repair capacity, and microenvironment-supported anti-apoptotic properties equip them to effectively withstand therapies designed to kill proliferating cells (King et al., 2017). In the context of immunotherapy, heterogeneity in antigen presentation machinery allows some clones to evade immune recognition. Concurrently, the spatial heterogeneity in the expression of immunosuppressive molecules and the infiltration of immune cells creates functional immune-privileged zones within the tumor. This results in a patchy distribution of effective immune responses, even when an immune reaction is present, leading to heterogeneous treatment efficacy.

#### 3.3.3 Emerging detection and dynamic monitoring strategies

To overcome the challenges that tumor heterogeneity poses to precision medicine, non-invasive dynamic monitoring technologies based on liquid biopsy and advanced imaging are rapidly developing. Circulating tumor DNA (ctDNA) analysis, which involves capturing and analyzing tumor-derived DNA fragments in the blood, offers the potential for a non-invasive “molecular biopsy” (Cescon et al., 2020). Its core value lies in overcoming the spatial limitations of single-site biopsies by providing an integrative reflection of the tumor’s global genomic landscape, potentially even revealing mutations undetected by tissue biopsy. More importantly, by tracking changes in variant allele frequencies and mutational profiles in longitudinal ctDNA samples, clinicians can monitor the dynamic evolution of different clonal subpopulations under therapeutic pressure in real-time and at a molecular level, thereby detecting early signs of resistance prior to radiographic progression.

However, the clinical translation of plasma-based ctDNA in GBM remains challenging compared to extracranial malignancies. The blood-brain barrier (BBB) acts as a physical barrier that restricts the shedding of tumor-derived DNA into the systemic circulation, often resulting in extremely low allele frequencies (Keller and Pantel, 2019). Furthermore, technical factors such as limited tumor volume, high levels of intratumoral necrosis, and suboptimal tumor-to-blood ratio often hinder the detection sensitivity of plasma ctDNA (Wu et al., 2025). Given these limitations, analysis of cerebrospinal fluid (CSF) ctDNA has emerged as a high-yield alternative. CSF, being in closer anatomical proximity to the tumor, allows for significantly higher detection rates and a more accurate reflection of the tumor’s mutational landscape (De Mattos-Arruda et al., 2015). While CSF sampling is more invasive, its superior sensitivity makes it an essential tool for monitoring localized recurrence.

Complementing DNA-based diagnostics, extracellular vesicles (EVs)—including exosomes—have emerged as a robust class of circulating biomarkers. Unlike ctDNA, which is often limited by low abundance in the blood, EVs protect their molecular cargo—including proteins, miRNAs, and mRNAs—from degradation, providing a stable and comprehensive molecular snapshot of the tumor (Verma et al., 2025). Moreover, EVs can traverse the BBB, rendering them highly accessible in peripheral biofluids and a valuable, non-invasive alternative for longitudinal monitoring.

Concurrently, radiomics provides a powerful, non-invasive tool for the assessment of intratumoral heterogeneity (Li et al., 2024). By extracting sub-visual textural features from standard medical images, radiomics indirectly reflects spatial variations, such as cellular density and necrotic extent. The integration of these machine learning-derived radiomic models with liquid biopsy data offers a holistic “monitoring triad”: this approach not only predicts key molecular markers (e.g., IDH status, MGMT methylation) but also assists in discriminating true tumor progression from treatment-related pseudo-progression, providing the necessary supplemental data for precise clinical decision-making.

#### 3.3.4 Novel therapeutic paradigms targeting tumor evolution

Confronted with the fundamental challenge of clonal evolution, the traditional “maximum kill” strategy aimed at eradication has revealed inherent limitations. Viewed through the lens of evolutionary biology, therapeutic resistance is an inevitable outcome of adaptive evolution in the tumor cell population under therapeutic selection pressure. Furthermore, the translation of evolutionary-informed therapies is hindered by the physiological barriers of the central nervous system, which severely restrict the intratumoral delivery of systemic agents.

**FIGURE 3:**
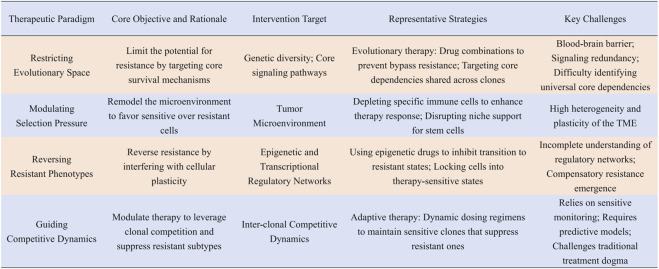
Evolution-informed therapeutic paradigms for targeting GBM tumor evolution. Summary of novel therapeutic strategies designed to manage rather than eradicate the GBM ecosystem. These paradigms shift the clinical focus from achieving maximal tumor kill to proactively steering the tumor’s evolutionary trajectory through niche modulation, phenotypic reversal, and competitive dynamics. Each approach is categorized by its core objective, intervention target, and inherent clinical challenges, providing a strategic roadmap for the future of dynamic precision medicine. Abbreviations: GBM, glioblastoma; TME, tumor microenvironment.

Addressing these delivery hurdles, engineering extracellular vesicles (EVs) offers a robust solution for CNS-targeted therapy. EVs can be bio-engineered to encapsulate specific therapeutic payloads and achieve targeted accumulation within the tumor niche, effectively overcoming the restrictions imposed by the blood-brain barrier (Verma et al., 2025). This strategy, coupled with advancements in standardized EV isolation and characterization, represents a critical frontier in developing precision, evolution-informed delivery platforms for GBM. Consequently, the core objective of novel therapeutic paradigms should shift from pursuing “maximum kill” towards “managing the evolutionary process.” The following four paradigms intervene at different levels of the tumor evolutionary process: (1) “Restricting Evolutionary Space” aims to reduce genetic diversity within the population; (2) “Modulating Selection Pressure” seeks to alter the microenvironment to confer a fitness advantage to sensitive over resistant cells; (3) “Reversing Resistant Phenotypes” aims to decrease the fitness of resistant phenotypes or increase the cost of their state transitions; and (4) “Guiding Competitive Dynamics” leverages natural competition between clones to suppress the expansion of resistant subpopulations. Together, these paradigms form a strategic framework with progressively deeper levels of intervention logic. To frame the following discussion, the core concepts, strategies, and challenges of these evolution-informed paradigms are systematically outlined in Figure 3.

##### 3.3.4.1 Restricting evolutionary space: evolutionary therapy and targeting core dependencies

This paradigm aims to constrain the evolutionary potential for resistance by attacking intrinsic survival and proliferation signaling pathways. Specific strategies include evolutionary therapy, which involves the prospective combination of agents targeting distinct survival mechanisms to prevent resistance driven by bypass signaling or compensatory pathways (West et al., 2020). For instance, in GBM models with EGFRvIII mutations, combining an EGFR inhibitor with a MET inhibitor can prevent resistance arising from compensatory MET pathway activation (Li et al., 2015). Another strategy is targeting core dependencies—attacking non-redundant molecular functions essential to all clonal subpopulations—with the goal of achieving universal cytotoxicity across all cellular subsets (Encarnación-Rosado et al., 2024).

##### 3.3.4.2 Modulating selection pressure: niche intervention

This paradigm does not directly attack tumor cells but indirectly steers evolutionary direction by modifying their supportive microenvironment. The intervention target is the TME, aiming to alter the selective pressures acting on tumor cells, thereby giving sensitive cells a fitness advantage over resistant ones. For example, using CSF-1R inhibitors to deplete tumor-associated macrophages can remodel the immune microenvironment in preclinical models, enhancing T-cell infiltration and overcoming resistance to checkpoint inhibitors (Foray et al., 2022). Similarly, targeting specific signaling molecules within the perivascular niche can undermine its support for GSCs stemness properties, thereby reducing their therapy resistance and regenerative potential (Ghochani et al., 2022).

##### 3.3.4.3 Reversing resistant phenotypes: targeting cellular state plasticity

The intervention target of this paradigm is the epigenetic regulation and state transition capacity of tumor cells. It aims to reverse resistant cells to a sensitive state or increase the cost of state switching by interfering with their plasticity. The principle involves targeting key epigenetic regulators that drive state transitions to guide or “lock” the cell population into specific functional states that are sensitive to therapy, thereby enhancing treatment efficacy (Shahani et al., 2025). For instance, a preclinical study demonstrated that HDAC inhibitors can suppress the transition of GBM cells to a mesenchymal state and potentially restore their sensitivity to temozolomide (Urdiciain et al., 2019).

##### 3.3.4.4 Guiding competitive dynamics: adaptive therapy

This paradigm targets the competitive dynamics within the tumor cell population. Its goal is not maximal kill but rather the fine modulation of therapeutic pressure to leverage sensitive cell subpopulations to suppress the expansion of resistant ones. This strategy, known as adaptive therapy, is often implemented by adjusting drug dosing or employing intermittent treatment schedules (West et al., 2020), aiming for long-term control of tumor burden rather than short-term eradication. While this approach has shown promise in early-phase clinical trials for other cancers like prostate cancer (Zhang et al., 2017), its successful implementation critically depends on sensitive dynamic monitoring technologies (e.g., liquid biopsy) to accurately quantify clonal dynamics, coupled with mathematical models to predict population competition outcomes under different dosing regimens.

##### 3.3.4.5 Practical challenges and translational barriers

Despite the promising prospects of the aforementioned evolution-informed therapeutic paradigms, their translation from concept to widespread clinical application faces a series of interconnected biological, technological, and clinical practice barriers. These systemic challenges constitute a formidable chasm that must be bridged to realize the vision of dynamic precision medicine.

At the biological and pharmacological level, the blood-brain barrier, unique to the central nervous system, presents a foremost obstacle. It severely restricts the effective intratumoral distribution of many macromolecular inhibitors, epigenetic drugs, and immunomodulators, rendering many promising systemic agents therapeutically irrelevant. Concurrently, the redundancy of tumor signaling pathways and the high unpredictability of evolutionary trajectories make the prospective design of effective “evolutionary therapy” combinations exceptionally difficult. Identifying broadly effective, druggable core dependencies shared across all clonal subpopulations remains a significant hurdle. Furthermore, the inherent heterogeneity and plasticity of the TME itself, coupled with an incomplete understanding of the full regulatory network governing cellular state plasticity, often results in limited efficacy of single interventions and the rapid emergence of compensatory mechanisms that undermine therapeutic impact.

On the technological and methodological front, a core challenge lies in the gap between theory and reality. However, these models are inevitably built upon simplifying assumptions, which limits their ability to fully recapitulate the complexity of the *in vivo* ecosystem, and their clinical predictive value urgently requires validation in prospective studies. Simultaneously, the “non-invasive molecular patrol”—such as high-frequency liquid biopsy and multi-omics analyses—that underpins dynamic precision medicine places severe demands on technological sensitivity, standardized protocols, and cost containment.

Regarding clinical and ethical considerations, this paradigm shift introduces fundamental challenges. Traditional clinical endpoints (e.g., progression-free survival) may not sensitively capture a treatment’s effect on the tumor evolutionary trajectory, necessitating the exploration of novel endpoints such as “evolutionary progression-free survival.” Strategies such as “adaptive therapy” aim to control the tumor rather than eradicate it. However, this goal of control conflicts with the deeply ingrained “maximum kill” mindset among both patients and physicians. Consequently, implementing such strategies requires thorough communication and consensus-building. Finally, establishing a closed-loop system of “diagnosis-treatment-monitoring-modeling-adaptation” imposes high demands on healthcare resources and overall systemic costs. Its accessibility and equity are practical concerns that must be addressed from the outset.

In summary, future progress will depend not on a single breakthrough, but on a concerted, multidisciplinary effort to address these interconnected challenges. The ultimate goal is to evolve the clinical management of GBM from a reactive pursuit into an intelligent process of dynamic control.

#### 3.3.5 Translating evolutionary paradigms into clinical trials

To translate evolutionary theory into actionable clinical trials, pilot studies should leverage dynamic biomarkers as exploratory endpoints to replace conventional “time-to-progression” metrics. For instance, in trials exploring niche modulation—such as combining CSF-1R inhibitors with standard radiotherapy (Pyonteck et al., 2013)—we propose a Phase I/II design where the primary endpoint is the Objective Response Rate (ORR), with exploratory longitudinal monitoring of immune infiltration and mesenchymal-like cell signatures in plasma (Rosenfeld et al., 2014). Similarly, to address reversing resistant phenotypes, a Phase I study evaluating HDAC inhibitors as sensitizers to temozolomide could utilize serial epigenetic profiling of ctDNA to establish the restoration of drug sensitivity as a secondary endpoint (Tehrani et al., 2025).

Furthermore, adaptive therapy—the most clinically advanced evolutionary paradigm—warrants an iterative Phase II design. Rather than fixed dosing, we propose an intermittent “evolutionary dosing” schedule, where treatment is paused or modulated based on real-time ctDNA variant allele frequencies (VAF). Here, the trial would utilize evolutionary progression-free survival (ePFS) as a surrogate marker of evolutionary control, defined as the interval before the emergence of resistant subclonal mutations (Nong et al., 2018). These designs, supported by recent evidence from longitudinal studies, provide a framework for future trials that treat the tumor not as a static entity, but as a dynamic, manageable system.

## 4 Discussion

The management paradigm for GBM is necessarily shifting from a reactive approach to a model of dynamic control. The in-depth dissection of tumor heterogeneity and clonal evolution reveals a fundamental clinical challenge: when the therapeutic target is a highly organized, continuously evolving ecosystem, traditional paradigms built upon static observations face profound limitations. Multi-omics evidence integrated in this review demonstrates that therapeutic resistance in GBM is an inevitable manifestation of its intrinsic biology under therapeutic selection pressure. This compels us to move beyond simplistic phenomenological summaries and critically re-evaluate our clinical framework to construct a path toward dynamic precision medicine.

The central dilemma in current practice stems from the tension between static diagnostic models and the dynamic nature of GBM. Diagnostic models reliant on single-site biopsies provide an incomplete picture of the tumor’s spatial architecture and evolutionary potential. Concurrently, “maximum kill” therapeutic strategies act as potent agents of natural selection, eliminating sensitive cells while providing a growth advantage for resistant clones. Consequently, intervening in this evolutionary dynamic has become a core factor directly influencing clinical efficacy.

Within this context, several interconnected academic controversies represent critical junctures for understanding and intervening in the tumor evolutionary process. The primary controversy centers on the dominant driver of therapeutic resistance: is it the selection of pre-existing resistant clones or therapy-induced adaptive cellular state switching? Existing evidence supports the coexistence and potential synergy of both models in GBM.

To effectively disentangle whether therapeutic resistance arises from the selection of pre-existing clones or therapy-induced transcriptional reprogramming, a multi-faceted experimental approach is required. As summarized in Table 1, current technologies offer distinct windows into these mechanisms. Lineage tracing and genetic barcoding provide the gold standard for tracking clonal fate at single-cell resolution (Suvà and Tirosh, 2020), while paired scDNA/scRNA-seq bridges the gap between fixed genetic alterations and plastic transcriptional states (Zhang et al., 2023; Weber et al., 2023). Furthermore, the integration of longitudinal ctDNA analysis with scRNA-seq facilitates the monitoring of evolutionary trajectories in clinical settings (Cescon et al., 2020; De Mattos-Arruda et al., 2015). Despite these advancements, each method carries inherent limitations—ranging from the high computational burden of multi-omics integration to the technical challenges of capturing rare, transient cellular states. Future studies must adopt an integrative strategy, leveraging these technologies to validate the spatiotemporal continuum of tumor adaptation.

**TABLE 1:** Methodological approaches to disentangle genetic selection from transcriptional plasticity.

Approach	Strengths	Limitations
Lineage tracing and barcoding	Enables real-time tracking of clonal fate and state transitions in vivo	Technically demanding; requires model system manipulation
Paired scDNA + scRNA-seq	Links genotype (clones) directly to phenotype (transcriptional states)	High cost; computational integration complexity
Longitudinal ctDNA + scRNA	Captures real-time evolutionary dynamics in patients	ctDNA low sensitivity; limited spatial context
Spatial multi-omics	Preserves spatial architecture of clonal niche	Lower throughput; complex tissue requirements

Longitudinal genomic studies reveal that recurrent clones are phylogenetically linked to specific ancestral subpopulations present at diagnosis. Simultaneously, single-cell transcriptomics confirms systematic reprogramming of the cellular state landscape following treatment, notably a marked enrichment towards a mesenchymal-like state. It is proposed that these two mechanisms may constitute a spatiotemporal continuum of adaptation: rapid plastic changes provide an initial survival buffer for the tumor population, while sustained therapeutic selection pressure ultimately fixes upon clones with genetic advantages. This understanding necessitates that future therapeutic strategies possess dual-targeting capabilities: specifically eliminating identified resistant clones while effectively intervening in the epigenetic regulatory networks that drive phenotypic switching.

Furthermore, we must carefully reconsider the dual role of therapeutic intervention itself in tumor evolution. Substantial evidence indicates that standard radiotherapy and chemotherapy, while cytotoxic, are themselves powerful sources of evolutionary pressure: radiotherapy can induce genomic instability, promoting new genetic variation; chemotherapy (e.g., temozolomide) can directly foster a hypermutator phenotype in a mismatch repair-deficient context; and both can shape an immunosuppressive microenvironment and induce pro-survival signaling, thereby creating a more supportive niche for resistant populations. This raises a crucial clinical consideration: do our current treatment strategies inadvertently create conditions conducive to a more malignant recurrence? Acknowledging this possibility is not to negate the clinical value of existing therapies but to emphasize the urgent need to develop strategies informed by ecological or evolutionary principles, exploring combinations of standard therapies with interventions that mitigate their pro-evolutionary effects. The goal shifts towards long-term disease control rather than short-term eradication, potentially leveraging competitive dynamics within the tumor population to suppress the expansion of resistant subclones.

Ultimately, all mechanistic insights and debate converge on a common translational goal: the transition from static classification to dynamic precision medicine. This paradigm shift relies on the synergistic establishment of three core pillars. First, the deep integration of technology and dynamic data acquisition. This requires the organic fusion of liquid biopsy, high-throughput sequencing, and advanced imaging biomarkers to establish clinical pathways for periodic molecular monitoring, enabling the non-invasive construction of personalized tumor evolutionary maps. Second, the systematic innovation of clinical trial design, requiring the creation and promotion of “evolution-informed” clinical trials, such as adaptive platform trials, capable of dynamically adjusting treatment strategies based on real-time molecular evolutionary data to directly test novel evolution-based interventions. Finally, the development and application of predictive mathematical models. By building computational models that integrate multi-omics data, microenvironmental features, and treatment history, we can transition from passive description to active prediction, identifying critical nodes driving evolution to enable pre-emptive therapeutic adjustments.

However, a critical awareness of the limitations inherent in the technological and theoretical frameworks supporting this new paradigm is essential. While single-cell and spatial multi-omics technologies provide unprecedented resolution, their application remains constrained by technical biases—for instance, the potential of scRNA-seq to overlook rare cell populations, and the current insufficient resolution of spatial transcriptomics for precisely delineating cell-cell interactions. Similarly, mathematical models designed to predict tumor evolutionary trajectories, though theoretically powerful, are built upon simplifying assumptions that struggle to fully capture the complex *in vivo* dynamics. Their clinical predictive value remains to be prospectively validated. Therefore, the paradigmatic revolution from static classification to dynamic ecological management, while conceptually the necessary path toward therapeutic breakthroughs, currently faces a significant chasm between theory and practical implementation at both technical and translational levels.

In summary, the discussion herein argues that conceptualizing GBM as a dynamically evolving ecosystem carries implications far beyond adding a new biological dimension. It fundamentally compels a re-examination of the ultimate goals and success metrics of therapeutic intervention. Translating this ecosystem model into actionable clinical practice stands as the central challenge before us.

## 5 Future perspectives

To bridge the gap between conceptual understanding and clinical implementation, future research and applications must converge on building a viable “Closed-Loop System for Dynamic Precision Medicine.” This system aims to transform GBM into a chronically managed disease through continuous sensing, intelligent decision-making, and adaptive intervention.

1. Seamless technological integration and clinical pathway innovation. The future foundation lies in advancing the standardization and streamlining of single-cell multi-omics, liquid biopsy, and radiomics technologies, followed by their seamless integration into routine clinical workflows. This will enable minimally invasive “molecular patrols,” facilitating the construction of personalized tumor evolutionary maps. Critically, this real-time data stream will directly support “evolution-informed” clinical trials, such as adaptive platform trials, designed to validate novel interventions based on evolutionary principles.
2. Synergistic advancement of mechanistic investigation and predictive modeling. Deeper dissection of core biological mechanisms, such as cellular state plasticity, must proceed in lockstep with the development of more reliable predictive models. Future computational models must evolve from descriptive tools into clinically valuable decision-support systems. This particularly requires the development and validation of artificial intelligence -driven approaches capable of integrating high spatiotemporal resolution longitudinal multi-omics and clinical data to simulate clonal dynamics and forecast intervention outcomes. These models must also incorporate key physiological constraints like the blood-brain barrier and drug pharmacokinetics to provide trustworthy simulations.
3. Overcoming central nervous system-specific barriers. The ultimate success of any intervention hinges on breakthroughs in innovative drug delivery strategies. Overcoming the blood-brain barrier must be placed at the core of systemic therapeutic development. Through strengthened interdisciplinary collaboration with biomaterials science and drug delivery engineering, priority should be given to developing small-molecule agents with superior brain penetrance or novel delivery platforms, ensuring that advanced therapeutics can effectively reach their target niches.
4. Proactive shaping of clinical paradigms and consensus building. Finally, and crucially, is the proactive shaping of new clinical paradigms. This involves collaborating with the medical community and patient communities to redefine treatment success metrics and build consensus around the goal of “long-term control rather than absolute eradication.” Patients must be educated about the rationale for dynamic precision management and engaged as active participants in the therapeutic decision-making loop. Only upon achieving this cognitive shift from “cure” to “management” can the closed-loop of dynamic precision medicine become fully operational.

By collaboratively advancing these four interdependent strategic fronts, we can systematically transform the clinical management of GBM from a protracted, reactive battle against tumor evolution into a dynamic control process capable of proactively sensing, predicting, and steering its evolutionary course.

## 6 Conclusion

Therapeutic resistance and recurrence in GBM stem from the intrinsic biological complexity of a highly heterogeneous and continuously evolving ecosystem. This review has delineated the manifestations of GBM heterogeneity across spatial and temporal dimensions, alongside the multi-layered mechanisms driving its clonal evolution. Collectively, these insights affirm that GBM must be monitored and targeted as a dynamic living system. The envisioned future of “dynamic precision medicine” lies in constructing a closed-loop system that integrates baseline multi-omics mapping, non-invasive molecular monitoring, model-assisted decision-making, and dynamic therapeutic adaptation. However, we must acknowledge that the realization of this vision remains a formidable challenge. Its path is hindered not only by the limitations of current technologies and theories discussed but also by practical barriers including the blood-brain barrier, established clinical practices, resource allocation, and ethical considerations. Future progress will depend not on a single breakthrough, but on deep interdisciplinary collaboration to systematically bridge these gaps while critically re-evaluating existing paradigms. Only through such a concerted effort can we transform the clinical management of GBM from a reactive pursuit into an intelligent process of proactive dynamic control.

## References

[B1] Alnahhas I. (2024). Molecular testing in gliomas: what is necessary in routine clinical practice? Curr. Oncol. Rep. 26 (11), 1277–1282. 10.1007/s11912-024-01602-w 39361075 PMC11579106

[B2] Bakr A. Della Corte G. Veselinov O. Kelekçi S. Chen M. M. Lin Y. Y. (2024). ARID1A regulates DNA repair through chromatin organization and its deficiency triggers DNA damage-mediated anti-tumor immune response. Nucleic Acids Res. 52 (10), 5698–5719. 10.1093/nar/gkae233 38587186 PMC11162808

[B3] Bhat K. P. L. Balasubramaniyan V. Vaillant B. Ezhilarasan R. Hummelink K. Hollingsworth F. (2013). Mesenchymal differentiation mediated by NF-κB promotes radiation resistance in glioblastoma. Cancer Cell. 24 (3), 331–346. 10.1016/j.ccr.2013.08.001 23993863 PMC3817560

[B4] Brennan C. W. Verhaak R. G. McKenna A. Campos B. Noushmehr H. Salama S. R. (2013). The somatic genomic landscape of glioblastoma. Cell. 155 (2), 462–477. 24120142 10.1016/j.cell.2013.09.034 PMC3910500

[B5] Calabrese C. Poppleton H. Kocak M. Hogg T. L. Fuller C. Hamner B. (2007). A perivascular niche for brain tumor stem cells. Cancer Cell. 11 (1), 69–82. 10.1016/j.ccr.2006.11.020 17222791

[B6] Caravagna G. Heide T. Williams M. J. Zapata L. Nichol D. Chkhaidze K. (2020). Subclonal reconstruction of tumors by using machine learning and population genetics. Nat. Genet. 52 (9), 898–907. 10.1038/s41588-020-0675-5 32879509 PMC7610388

[B7] Cescon D. W. Bratman S. V. Chan S. M. Siu L. L. (2020). Circulating tumor DNA and liquid biopsy in oncology. Nat. Cancer 1 (3), 276–290. 10.1038/s43018-020-0043-5 35122035

[B8] Chabon J. J. Simmons A. D. Lovejoy A. F. Esfahani M. S. Newman A. M. Haringsma H. J. (2016). Circulating tumour DNA profiling reveals heterogeneity of EGFR inhibitor resistance mechanisms in lung cancer patients. Nat. Commun. 7, 11815. 10.1038/ncomms11815 27283993 PMC4906406

[B9] Chandra A. Jahangiri A. Chen W. Nguyen A. T. Yagnik G. Pereira M. P. (2020). Clonal ZEB1-Driven mesenchymal transition promotes targetable oncologic antiangiogenic therapy resistance. Cancer Res. 80 (7), 1498–1511. 10.1158/0008-5472.CAN-19-1305 32041837 PMC7236890

[B10] Chang C. Chavarro V. S. Gerstl J. V. E. Blitz S. E. Spanehl L. Dubinski D. (2024). Recurrent glioblastoma-molecular underpinnings and evolving treatment paradigms. Int. J. Mol. Sci. 25 (12), 6733. 10.3390/ijms25126733 38928445 PMC11203521

[B11] Cooper A. J. Sequist L. V. Lin J. J. (2022). Third-generation EGFR and ALK inhibitors: mechanisms of resistance and management. Nat. Rev. Clin. Oncol. 19 (8), 499–514. 10.1038/s41571-022-00639-9 35534623 PMC9621058

[B12] Couturier C. P. Ayyadhury S. Le P. U. Nadaf J. Monlong J. Riva G. (2020). Single-cell RNA-Seq reveals that glioblastoma recapitulates a normal neurodevelopmental hierarchy. Nat. Commun. 11 (1), 3406. 10.1038/s41467-020-17186-5 32641768 PMC7343844

[B13] Crespo I. Vital A. L. Gonzalez-Tablas M. Patino Mdel C. Otero A. Lopes M. C. (2015). Molecular and genomic alterations in glioblastoma multiforme. Am. J. Pathol. 185 (7), 1820–1833. 10.1016/j.ajpath.2015.02.023 25976245

[B14] Cui X. Morales R. T. Qian W. Wang H. Gagner J. P. Dolgalev I. (2018). Hacking macrophage-associated immunosuppression for regulating glioblastoma angiogenesis. Biomaterials 161, 164–178. 10.1016/j.biomaterials.2018.01.053 29421553 PMC8059366

[B15] De Mattos-Arruda L. Mayor R. Ng C. K. Y. Weigelt B. Martínez-Ricarte F. Torrejon D. (2015). Cerebrospinal fluid-derived circulating tumour DNA better represents the genomic alterations of brain tumours than plasma. Nat. Commun. 6, 8839. 10.1038/ncomms9839 26554728 PMC5426516

[B16] Eisenbarth D. Wang Y. A. (2023). Glioblastoma heterogeneity at single cell resolution. Oncogene 42 (27), 2155–2165. 10.1038/s41388-023-02738-y 37277603 PMC10913075

[B17] Encarnación-Rosado J. Sohn A. S. W. Biancur D. E. Lin E. Y. Osorio-Vasquez V. Rodrick T. (2024). Targeting pancreatic cancer metabolic dependencies through glutamine antagonism. Nat. Cancer 5 (1), 85–99. 10.1038/s43018-023-00647-3 37814010 PMC10824664

[B18] Espinoza F. I. Tankov S. Chliate S. Pereira Couto J. Marinari E. Vermeil T. (2025). Targeting HIF-2α in glioblastoma reshapes the immune infiltrate and enhances response to immune checkpoint blockade. Cell. Mol. Life Sci. 82 (1), 119. 10.1007/s00018-025-05642-8 40095115 PMC11914682

[B19] Favero F. McGranahan N. Salm M. Birkbak N. J. Sanborn J. Z. Benz S. C. (2015). Glioblastoma adaptation traced through decline of an IDH1 clonal driver and macro-evolution of a double-minute chromosome. Ann. Oncol. 26 (5), 880–887. 10.1093/annonc/mdv127 25732040 PMC4405282

[B20] Foray C. Barca C. Winkeler A. Wagner S. Hermann S. Schäfers M. (2022). Interrogating glioma-associated microglia and macrophage dynamics under CSF-1R therapy with multitracer *in vivo* PET/MRI. J. Nucl. Med. 63 (9), 1386–1393. 10.2967/jnumed.121.263318 35115369 PMC9454459

[B21] Garner J. M. Fan M. Yang C. H. Du Z. Sims M. Davidoff A. M. (2013). Constitutive activation of signal transducer and activator of transcription 3 (STAT3) and nuclear factor κB signaling in glioblastoma cancer stem cells regulates the notch pathway. J. Biol. Chem. 288 (36), 26167–26176. 10.1074/jbc.M113.477950 23902772 PMC3764819

[B22] Gerlinger M. Rowan A. J. Horswell S. Math M. Larkin J. Endesfelder D. (2012). Intratumor heterogeneity and branched evolution revealed by multiregion sequencing. N. Engl. J. Med. 366 (10), 883–892. 10.1056/NEJMoa1113205 22397650 PMC4878653

[B23] Ghochani Y. Muthukrishnan S. D. Sohrabi A. Kawaguchi R. Condro M. C. Bastola S. (2022). A molecular interactome of the glioblastoma perivascular niche reveals integrin binding sialoprotein as a mediator of tumor cell migration. Cell. Rep. 41 (3), 111511. 10.1016/j.celrep.2022.111511 36261010 PMC9642966

[B24] Goenka A. Tiek D. Song X. Huang T. Hu B. Cheng S. Y. (2021). The many facets of therapy resistance and tumor recurrence in glioblastoma. Cells 10 (3), 484. 10.3390/cells10030484 33668200 PMC7995978

[B25] Hadad S. Gupta R. Oberheim Bush N. A. Taylor J. W. Villanueva-Meyer J. E. Young J. S. (2023). *De novo* replication repair deficient glioblastoma, IDH-wildtype is a distinct glioblastoma subtype in adults that May benefit from immune checkpoint blockade. Acta Neuropathol. 147 (1), 3. 10.1007/s00401-023-02654-1 38079020 PMC10713691

[B26] Hara T. Chanoch-Myers R. Mathewson N. D. Myskiw C. Atta L. Bussema L. (2021). Interactions between cancer cells and immune cells drive transitions to mesenchymal-like states in glioblastoma. Cancer Cell. 39 (6), 779. 10.1016/j.ccell.2021.05.002 34087162 PMC8366750

[B27] Hart W. S. Myers P. J. Purow B. W. Lazzara M. J. (2024). Divergent transcriptomic signatures from putative mesenchymal stimuli in glioblastoma cells. Cancer Gene Ther. 31 (6), 851–860. 10.1038/s41417-023-00724-w 38337036 PMC11192628

[B28] Hegi M. E. Diserens A. C. Gorlia T. Hamou M. F. de Tribolet N. Weller M. (2005). MGMT gene silencing and benefit from temozolomide in glioblastoma. N. Engl. J. Med. 352 (10), 997–1003. 10.1056/NEJMoa043331 15758010

[B29] Hendriksen J. D. Locallo A. Maarup S. Debnath O. Ishaque N. Hasselbach B. (2024). Immunotherapy drives mesenchymal tumor cell state shift and TME immune response in glioblastoma patients. Neuro Oncol. 26 (8), 1453–1466. 10.1093/neuonc/noae085 38695342 PMC11300009

[B30] Johnson B. E. Mazor T. Hong C. Barnes M. Aihara K. McLean C. Y. (2014). Mutational analysis reveals the origin and therapy-driven evolution of recurrent glioma. Science 343 (6167), 189–193. 10.1126/science.1239947 24336570 PMC3998672

[B31] Kampers L. F. C. Metselaar D. S. Vinci M. Scirocchi F. Veldhuijzen van Zanten S. Eyrich M. (2025). The complexity of malignant glioma treatment. Cancers (Basel) 17 (5), 879. 10.3390/cancers17050879 40075726 PMC11899524

[B32] Keller L. Pantel K. (2019). Unravelling tumour heterogeneity by single-cell profiling of circulating tumour cells. Nat. Rev. Cancer 19 (10), 553–567. 10.1038/s41568-019-0180-2 31455893

[B33] Kim H. Zheng S. Amini S. S. Virk S. M. Mikkelsen T. Brat D. J. (2015). Whole-genome and multisector exome sequencing of primary and post-treatment glioblastoma reveals patterns of tumor evolution. Genome Res. 25 (3), 316–327. 10.1101/gr.180612.114 25650244 PMC4352879

[B34] King H. O. Brend T. Payne H. L. Wright A. Ward T. A. Patel K. (2017). RAD51 is a selective DNA repair target to radiosensitize glioma stem cells. Stem Cell. Rep. 8 (1), 125–139. 10.1016/j.stemcr.2016.12.005 PMC5233453 28076755

[B35] Lai Y. Lu X. Liao Y. Ouyang P. Wang H. Zhang X. (2024). Crosstalk between glioblastoma and tumor microenvironment drives proneural-mesenchymal transition through ligand-receptor interactions. Genes. Dis. 11 (2), 874–889. 10.1016/j.gendis.2023.05.025 37692522 PMC10491977

[B36] Lemoine C. Da Veiga M. A. Rogister B. Piette C. Neirinckx V. (2025). An integrated perspective on single-cell and spatial transcriptomic signatures in high-grade gliomas. NPJ Precis. Oncol. 9 (1), 44. 10.1038/s41698-025-00830-y 39934275 PMC11814291

[B37] Li Z. Bao S. Wu Q. Wang H. Eyler C. Sathornsumetee S. (2009). Hypoxia-inducible factors regulate tumorigenic capacity of glioma stem cells. Cancer Cell. 15 (6), 501–513. 10.1016/j.ccr.2009.03.018 19477429 PMC2693960

[B38] Li L. Puliyappadamba V. T. Chakraborty S. Rehman A. Vemireddy V. Saha D. (2015). EGFR wild type antagonizes EGFRvIII-mediated activation of met in glioblastoma. Oncogene 34 (1), 129–134. 10.1038/onc.2013.534 24362532 PMC4804705

[B39] Li L. Xiao F. Wang S. Kuang S. Li Z. Zhong Y. (2024). Preoperative prediction of MGMT promoter methylation in glioblastoma based on multiregional and multi-sequence MRI radiomics analysis. Sci. Rep. 14 (1), 16031. 10.1038/s41598-024-66653-2 38992201 PMC11239670

[B40] Lin H. Liu C. Hu A. Zhang D. Yang H. Mao Y. (2024). Understanding the immunosuppressive microenvironment of glioma: mechanistic insights and clinical perspectives. J. Hematol. Oncol. 17 (1), 31. 10.1186/s13045-024-01544-7 38720342 PMC11077829

[B41] Liu M. Ji Z. Jain V. Smith V. L. Hocke E. Patel A. P. (2024a). Spatial transcriptomics reveals segregation of tumor cell states in glioblastoma and marked immunosuppression within the perinecrotic niche. Acta Neuropathol. Commun. 12 (1), 64. 10.1186/s40478-024-01769-0 38650010 PMC11036705

[B42] Liu Y. Zhou F. Ali H. Lathia J. D. Chen P. (2024b). Immunotherapy for glioblastoma: current state, challenges, and future perspectives. Cell. Mol. Immunol. 21 (12), 1354–1375. 10.1038/s41423-024-01226-x 39406966 PMC11607068

[B43] Loftus A. E. P. Romano M. S. Phuong A. N. McKinnel B. J. Muir M. T. Furqan M. (2024). An ILK/STAT3 pathway controls glioblastoma stem cell plasticity. Dev. Cell. 59 (24), 3197. 10.1016/j.devcel.2024.09.003 39326421

[B44] Long G. V. Shklovskaya E. Satgunaseelan L. Mao Y. da Silva I. P. Perry K. A. (2025). Neoadjuvant triplet immune checkpoint blockade in newly diagnosed glioblastoma. Nat. Med. 31 (5), 1557–1566. 10.1038/s41591-025-03512-1 40016450 PMC12092302

[B45] Lu J. Huo W. Ma Y. Wang X. Yu J. (2024). Suppressive immune microenvironment and CART therapy for glioblastoma: future prospects and challenges. Cancer Lett. 600, 217185. 10.1016/j.canlet.2024.217185 39142498

[B46] Lv S. Q. Fu Z. Yang L. Li Q. R. Zhu J. Gai Q. J. (2022). Comprehensive omics analyses profile genesets related with tumor heterogeneity of multifocal glioblastomas and reveal LIF/CCL2 as biomarkers for mesenchymal subtype. Theranostics 12 (1), 459–473. 10.7150/thno.65739 34987659 PMC8690928

[B47] Mahajan K. Mahajan N. P. (2015). Cross talk of tyrosine kinases with the DNA damage signaling pathways. Nucleic Acids Res. 43 (22), 10588–10601. 10.1093/nar/gkv1166 26546517 PMC4678820

[B48] Mathur R. Wang Q. Schupp P. G. Nikolic A. Hilz S. Hong C. (2024). Glioblastoma evolution and heterogeneity from a 3D whole-tumor perspective. Cell. 187 (2), 446. 10.1016/j.cell.2023.12.013 38242087 PMC10832360

[B49] Matsumoto Y. Ichikawa T. Kurozumi K. Otani Y. Fujimura A. Fujii K. (2020). Annexin A2-STAT3-Oncostatin M receptor axis drives phenotypic and mesenchymal changes in glioblastoma. Acta Neuropathol. Commun. 8 (1), 42. 10.1186/s40478-020-00916-7 32248843 PMC7132881

[B50] Minea R. O. Thein T. Z. Yang Z. Campan M. Ward P. M. Schönthal A. H. (2024). NEO212, temozolomide conjugated to NEO100, exerts superior therapeutic activity over temozolomide in preclinical chemoradiation models of glioblastoma. Neurooncol Adv. 6 (1), vdae095. 10.1093/noajnl/vdae095 39022643 PMC11252566

[B51] Moujalled D. Southon A. G. Saleh E. Brinkmann K. Ke F. Iliopoulos M. (2022). BH3 mimetic drugs cooperate with temozolomide, JQ1 and inducers of ferroptosis in killing glioblastoma multiforme cells. Cell. Death Differ. 29 (7), 1335–1348. 10.1038/s41418-022-00977-2 35332309 PMC9287558

[B52] Nomura M. Spitzer A. Johnson K. C. Garofano L. Nehar-Belaid D. Galili Darnell N. (2025). The multilayered transcriptional architecture of glioblastoma ecosystems. Nat. Genet. 57 (5), 1155–1167. 10.1038/s41588-025-02167-5 40346361 PMC12081307

[B53] Nong J. Gong Y. Guan Y. Yi X. Yi Y. Chang L. (2018). Circulating tumor DNA analysis depicts subclonal architecture and genomic evolution of small cell lung cancer. Nat. Commun. 9 (1), 3114. 10.1038/s41467-018-05327-w 30082701 PMC6079068

[B54] Onubogu U. Gatenbee C. D. Prabhakaran S. Wolfe K. L. Oakes B. Salatino R. (2024). Spatial analysis of recurrent glioblastoma reveals perivascular niche organization. JCI Insight 9 (12), e179853. 10.1172/jci.insight.179853 38805346 PMC11383164

[B55] Pu J. Yuan K. Tao J. Qin Y. Li Y. Fu J. (2025). Glioblastoma multiforme: an updated overview of temozolomide resistance mechanisms and strategies to overcome resistance. Discov. Oncol. 16 (1), 731. 10.1007/s12672-025-02567-3 40353925 PMC12069213

[B56] Pyonteck S. M. Akkari L. Schuhmacher A. J. Bowman R. L. Sevenich L. Quail D. F. (2013). CSF-1R inhibition alters macrophage polarization and blocks glioma progression. Nat. Med. 19 (10), 1264–1272. 10.1038/nm.3337 24056773 PMC3840724

[B57] Qazi M. A. Salim S. K. Brown K. R. Mikolajewicz N. Savage N. Han H. (2022). Characterization of the minimal residual disease state reveals distinct evolutionary trajectories of human glioblastoma. Cell. Rep. 40 (13), 111420. 10.1016/j.celrep.2022.111420 36170831

[B58] Read R. D. Tapp Z. M. Rajappa P. Hambardzumyan D. (2024). Glioblastoma microenvironment-from biology to therapy. Genes. Dev. 38 (9-10), 360–379. 10.1101/gad.351427.123 38811170 PMC11216181

[B59] Rosenfeld M. R. Ye X. Supko J. G. Desideri S. Grossman S. A. Brem S. (2014). A phase I/II trial of hydroxychloroquine in conjunction with radiation therapy and concurrent and adjuvant temozolomide in patients with newly diagnosed glioblastoma multiforme. Autophagy 10 (8), 1359–1368. 10.4161/auto.28984 24991840 PMC4203513

[B60] Setlai B. P. Hull R. Reis R. M. Agbor C. Ambele M. A. Mulaudzi T. V. (2022). MicroRNA interrelated epithelial mesenchymal transition (EMT) in glioblastoma. Genes. (Basel) 13 (2), 244. 10.3390/genes13020244 35205289 PMC8872331

[B61] Shahani A. Slika H. Elbeltagy A. Lee A. Peters C. Dotson T. (2025). The epigenetic mechanisms involved in the treatment resistance of glioblastoma. Cancer Drug Resist 8, 12. 10.20517/cdr.2024.157 40201311 PMC11977385

[B62] Shi P. Xu J. Cui H. (2023). The recent research progress of NF-κB signaling on the proliferation, migration, invasion, immune escape and drug resistance of glioblastoma. Int. J. Mol. Sci. 24 (12), 10337. 10.3390/ijms241210337 37373484 PMC10298967

[B63] Singh N. Miner A. Hennis L. Mittal S. (2021). Mechanisms of temozolomide resistance in glioblastoma - a comprehensive review. Cancer Drug Resist 4 (1), 17–43. 34337348 10.20517/cdr.2020.79 PMC8319838

[B64] Snuderl M. Fazlollahi L. Le L. P. Nitta M. Zhelyazkova B. H. Davidson C. J. (2011). Mosaic amplification of multiple receptor tyrosine kinase genes in glioblastoma. Cancer Cell. 20 (6), 810–817. 10.1016/j.ccr.2011.11.005 22137795

[B65] Sottoriva A. Spiteri I. Piccirillo S. G. Touloumis A. Collins V. P. Marioni J. C. (2013). Intratumor heterogeneity in human glioblastoma reflects cancer evolutionary dynamics. Proc. Natl. Acad. Sci. U. S. A. 110 (10), 4009–4014. 10.1073/pnas.1219747110 23412337 PMC3593922

[B66] Suvà M. L. Tirosh I. (2020). The glioma stem cell model in the era of single-cell genomics. Cancer Cell. 37 (5), 630–636. 10.1016/j.ccell.2020.04.001 32396858

[B67] Tehrani G. A. Kubick R. N. Zarodniuk M. Datta M. (2025). Histone deacetylase inhibitors sensitize glioblastoma models to temozolomide and reprogram immunosuppressive myeloid cells. Sci. Rep. 15 (1), 36804. 10.1038/s41598-025-20749-5 41120507 PMC12540765

[B68] Urdiciain A. Erausquin E. Meléndez B. Rey J. A. Idoate M. A. Castresana J. S. (2019). Tubastatin A, an inhibitor of HDAC6, enhances temozolomide-induced apoptosis and reverses the malignant phenotype of glioblastoma cells. Int. J. Oncol. 54 (5), 1797–1808. 10.3892/ijo.2019.4739 30864703

[B69] Verhaak R. G. Hoadley K. A. Purdom E. Wang V. Qi Y. Wilkerson M. D. (2010). Integrated genomic analysis identifies clinically relevant subtypes of glioblastoma characterized by abnormalities in PDGFRA, IDH1, EGFR, and NF1. Cancer Cell. 17 (1), 98–110. 10.1016/j.ccr.2009.12.020 20129251 PMC2818769

[B70] Verma N. Franchitto M. Zonfrilli A. Cialfi S. Palermo R. Talora C. (2019). DNA damage stress: cui prodest? Int. J. Mol. Sci. 20 (5). 10.3390/ijms20051073 30832234 PMC6429504

[B71] Verma N. Arora S. Singh A. K. Ahmed J. (2025). Unlocking the potential of exosomes ‘extracellular vesicles’: drug delivery advancements and therapeutics in ocular diseases. RSC Pharm. 2 (6), 1201–1226. 10.1039/d5pm00097a

[B72] Vitiello P. P. Rousseau B. Chilà R. Battuello P. Amodio V. Battaglieri V. (2025). Cisplatin and temozolomide combinatorial treatment triggers hypermutability and immune surveillance in experimental cancer models. Cancer Cell. 43 (7), 1296. 10.1016/j.ccell.2025.05.014 40513578

[B73] Wang L. Jung J. Babikir H. Shamardani K. Jain S. Feng X. (2022). A single-cell atlas of glioblastoma evolution under therapy reveals cell-intrinsic and cell-extrinsic therapeutic targets. Nat. Cancer 3 (12), 1534–1552. 10.1038/s43018-022-00475-x 36539501 PMC9767870

[B74] Weber L. L. Zhang C. Ochoa I. El-Kebir M. (2023). Phertilizer: growing a clonal tree from ultra-low coverage single-cell DNA sequencing of tumors. PLoS Comput. Biol. 19 (10), e1011544. 10.1371/journal.pcbi.1011544 37819942 PMC10593221

[B75] West J. You L. Zhang J. Gatenby R. A. Brown J. S. Newton P. K. (2020). Towards multidrug adaptive therapy. Cancer Res. 80 (7), 1578–1589. 10.1158/0008-5472.CAN-19-2669 31948939 PMC7307613

[B76] Winkler F. (2023). The winner takes it all: competition drives clonal selection in gliomagenesis. Cancer Cell. 41 (8), 1394–1396. 10.1016/j.ccell.2023.07.003 37541246

[B77] Wu Q. Wu J. Wang F. Wang Q. Dai W. Yang Y. (2025). Chromatin accessibility derived from cfDNA serves as a novel classification biomarker of glioma. Front. Oncol. 15, 1688625. 10.3389/fonc.2025.1688625 41473442 PMC12745158

[B78] Xie H. Jiang Y. Xiang Y. Wu B. Zhao J. Huang R. (2024). Super-enhancer-driven LIF promotes the mesenchymal transition in glioblastoma by activating ITGB2 signaling feedback in microglia. Neuro Oncol. 26 (8), 1438–1452. 10.1093/neuonc/noae065 38554116 PMC11300025

[B79] Xiong W. Liao Y. Qin J. Y. Li W. H. Tang Z. Y. (2020). Adverse effects of chemoradiotherapy on invasion and metastasis of tumor cells. Genes. Dis. 7 (3), 351–358. 10.1016/j.gendis.2020.04.004 32884989 PMC7452502

[B80] Yang F. Akhtar M. N. Zhang D. El-Mayta R. Shin J. Dorsey J. F. (2024). An immunosuppressive vascular niche drives macrophage polarization and immunotherapy resistance in glioblastoma. Sci. Adv. 10 (9), eadj4678. 10.1126/sciadv.adj4678 38416830 PMC10901371

[B81] Zhang J. Cunningham J. J. Brown J. S. Gatenby R. A. (2017). Integrating evolutionary dynamics into treatment of metastatic castrate-resistant prostate cancer. Nat. Commun. 8 (1), 1816. 10.1038/s41467-017-01968-5 29180633 PMC5703947

[B82] Zhang X. Wang X. Xu R. Ji J. Xu Y. Han M. (2018). YM155 decreases radiation-induced invasion and reverses epithelial-mesenchymal transition by targeting STAT3 in glioblastoma. J. Transl. Med. 16 (1), 79. 10.1186/s12967-018-1451-5 29571296 PMC5865331

[B83] Zhang B. Chen Y. Shi X. Zhou M. Bao L. Hatanpaa K. J. (2021). Regulation of branched-chain amino acid metabolism by hypoxia-inducible factor in glioblastoma. Cell. Mol. Life Sci. 78 (1), 195–206. 32088728 10.1007/s00018-020-03483-1 PMC8112551

[B84] Zhang L. Bass H. W. Irianto J. Mallory X. (2023). Integrating SNVs and CNAs on a phylogenetic tree from single-cell DNA sequencing data. Genome Res. 33 (11), 2002–2017. 10.1101/gr.277249.122 37993137 PMC10760445

[B85] Zhu X. Liu S. Yang X. Wang W. Shao W. Ji T. (2021). P4HA1 as an unfavorable prognostic marker promotes cell migration and invasion of glioblastoma via inducing EMT process under hypoxia microenvironment. Am. J. Cancer Res. 11 (2), 590–617. 33575089 PMC7868758

