# Supplementary figures and images for: The glioblastoma ecosystem: clonal evolution, heterogeneity, and therapeutic resistance
Source: Front Cell Dev Biol. 2026 Jul 1;14:1834106. doi: 10.3389/fcell.2026.1834106 (PMC13368775; doi:10.3389/fcell.2026.1834106)

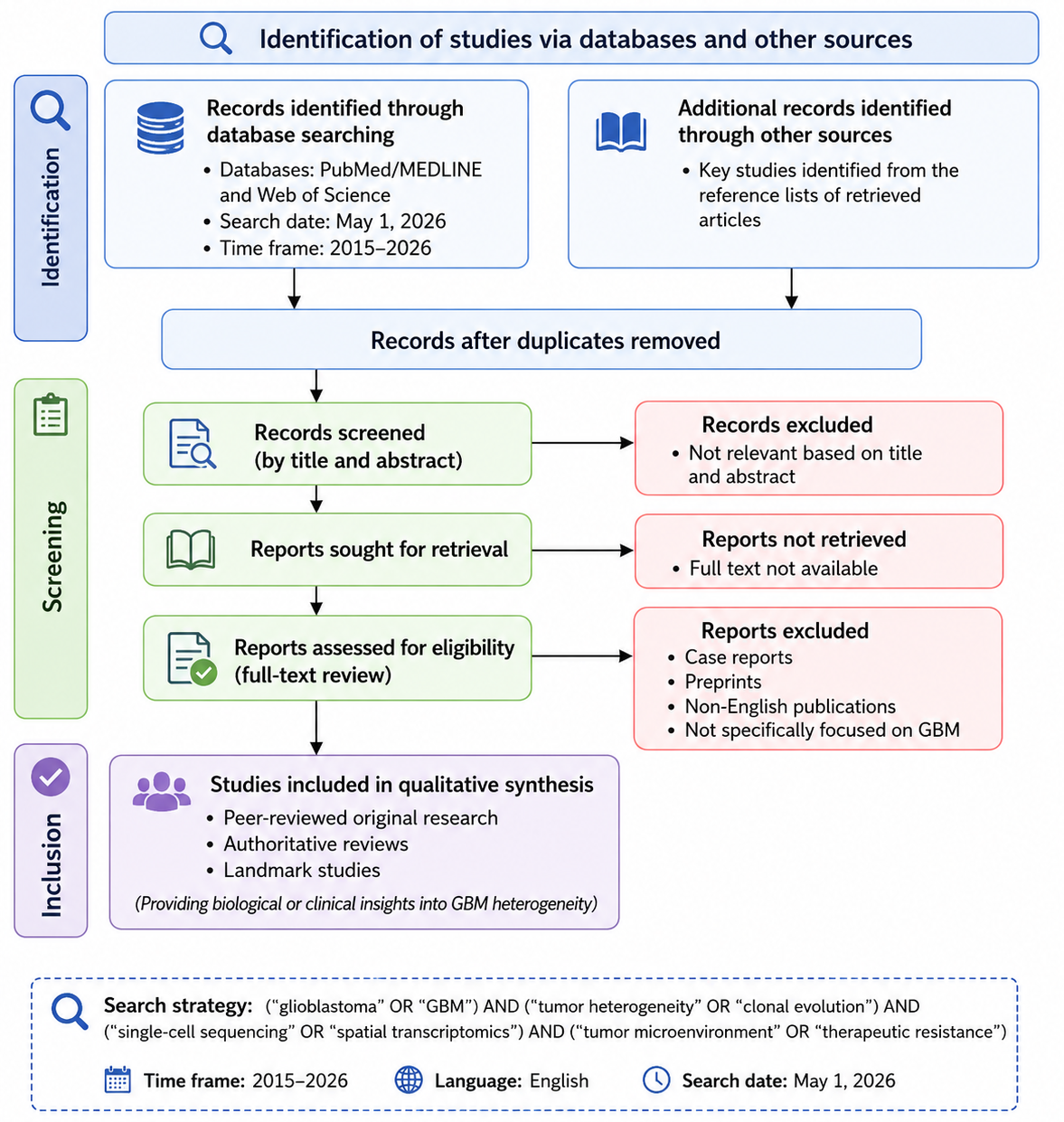

Supplement: Supplementary file 1 [file Image1.TIF]
